# MAGIC- is it for real?

**DOI:** 10.1186/1546-0096-13-S1-P156

**Published:** 2015-09-28

**Authors:** L Damian, M Velcherean, M Andrei, I Felea, P Vele, S Rednic

**Affiliations:** 1 Emergency Clinical County Hospital Cluj, Rheumatology, Cluj-Napoca, Romania; 2 Emergency County Hospital Deva, Rheumatology, Deva, Romania; 3 “Iuliu Hatieganu” University of Medicne and Pharmacy Cluj, Rheumatology, Cluj-Napoca, Romania

## Question

MAGIC syndrome, an acronym for mouth and genital ulcers with inflammed cartilage, is a rare condition described in 1985 [1]. About 20 cases have been reported [2], and its existence is challenged, as it could be just a mere association of Bechet's disease (BD) with relapsing polychondritis (RP) [3]. Other authors, however, consider it a distinct entity with higher risk of aortic aneurysms [4]. We tried to find out whether this syndrome is a true nosologic entity.

## Methods

We retrospectively reviewed our tertiary referral centre's database from 2000 to 2015 in order to identify the cases of RP and BD. All patients fulfilled the International Criteria for BD [5] and the Damiani-Levine criteria for RP [6].

## Results

Three cases have been identified, all diagnosed with MAGIC's syndrome since the first presentation. No other case evolved into MAGIC after an initial diagnosis of RP or BD. Hematological screening was negative in all patients; one had gastrointestinal vasculitis and another one panniculitis. No one in our series had eye or CNS involvement. Aortic aneurysms were absent (as yet) in the 2 patients searched for. Azathioprine, colchicine and corticotherapy were employed effectively in all patients.

**Table 1:** Clinical features of MAGIC patients in our series

Case	Sex, age	Clinical features	Therapy	Aortic involvement	Outcome
1	F, 63	oral aphtae since youth; genital aphtae, deep vein thrombosis, migratory seronegative polyarthritis, reccurent bilateral auricular chondritis, wheesing	CS, AZA,Col	NK	Lost to follow-up (after 2 years)

2	F, 35	bipolar aphtae, asymmetric sacroiliitis, acneiform rash, intermittent seronegative polyarthritis, gastrointestinal involvement, bilateral auricular and nasal chondritis ANA positive, dsDNA negative	CS, AZA,Col	No/NK	Lost to follow-up (after 3 years- emigrated)
3	M, 2	bipolar aphtae, pseudofolliculitis, erythema nodosum, arthritis, panniculitis, recurrent auricular chondritis, nasal chondritis, positive cartilage biopsy	CS, AZA,Col	No	Rare chondritis flares

CS=corticosteroids, Col=colchicine, AZA=azathioprine

## Conclusion

RP and BD have overlapping features and may share pathogenetic mechanisms. The same time of onset of the main MAGIC clinical features could favour the classification of the disease as distinct from RP and BD alone. However, in our small and incompletely followed-up series aortic aneurysms were not seen, like in other MAGIC cases reported. An aortic follow-up is nevertheless advisable, as in any RP.
